# The paradox of senescent-marker positive cancer cells: challenges and opportunities

**DOI:** 10.1038/s41514-024-00168-y

**Published:** 2024-09-14

**Authors:** Emily A. O’Sullivan, Ryan Wallis, Federica Mossa, Cleo L. Bishop

**Affiliations:** Blizard Institute, Barts and The London School of Medicine and Dentistry, Queen Mary University of London, London, UK

**Keywords:** Senescence, Cancer

## Abstract

Senescence is an anti-tumour mechanism and hallmark of cancer. Loss or mutation of key senescence effectors, such as p16INK4A, are frequently observed in cancer. Intriguingly, some human tumours are both proliferative and senescent-marker positive (Sen-Mark+). Here, we explore this paradox, focusing on the prognostic consequences and the current challenges in classifying these cells. We discuss future strategies for Sen-Mark+ cell detection together with emerging opportunities to exploit senescence for cancer.

## Introduction

Senescence is a homeostatic cellular programme that is activated in response to a plethora of stressors. In response, Cyclin Dependent Kinase inhibitors (CDKi), such as p16^INK4a^ (p16; encoded by the CDKN2A locus) and/or p21^WAF-1/CAP1^ (p21), are upregulated to initiate and maintain a stable cell cycle arrest. A further defining feature of senescence is an altered, hypersecretory phenotype, the composition of which is both trigger-dependent and temporally dynamic. The senescence-associated secretory phenotype (SASP) can contain a myriad of components, including proteins (interleukins, cytokines, chemokines, growth factors, proteases etc.), small extracellular vesicles, bioactive lipids and non-coding nucleic acids (e.g. miRNAs, cytoplasmic chromatin fragments) (reviewed in ref. ^1^). Emerging evidence suggests that the physiological setting governs whether the paracrine effect of the SASP has a beneficial or detrimental consequences for the local tissue microenvironment (TME).

Oncogene-induced senescence (OIS) is an established form of premature senescence that can be triggered following oncogene activation or tumour suppressor inactivation. Once established, OIS acts to prevent the replication of damaged cells at risk of malignant transformation, and is, therefore, considered a key tumour suppressor mechanism. Although not fully elaborated mechanistically, senescence escape refers to the re-acquisition of proliferative potential following senescence induction (reviewed in ref. ^2^). Subsequent, successive rounds of error-prone replication in these pre-cancerous cells can lead to the accumulation of more DNA damage and increased genomic instability, thereby allowing further pro-tumourigenic mutations to take place^3^. Thus, cancer evolution can result in proliferative tumour cells harbouring mutations in key senescence effector molecules. However, contrarily, some tumours are able to proliferate whilst also expressing high levels of key senescence effector proteins. These enigmatic ‘Senescent Marker Positive’ (Sen-Mark+) cancer cells have, to date, received comparatively little attention.

**Fig. 1:**
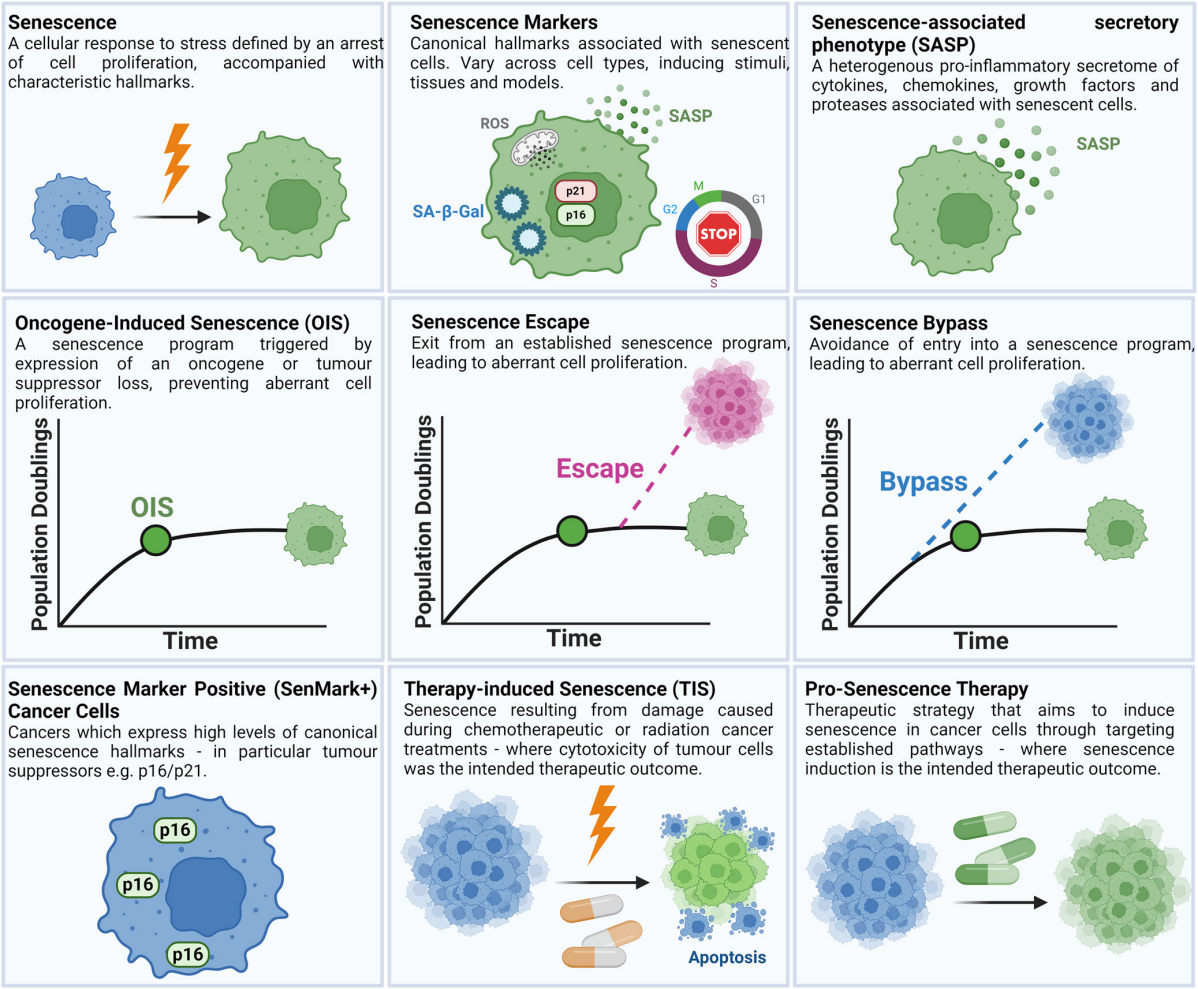
The definitions of key senescence-related processes. This figure provides definitions with diagrammatic examples of key senescence-related processes used within this review.

In this review, we chart the discovery of OIS and summarise the role of senescence as an intrinsic tumour suppressor mechanism. We outline examples where non-malignant senescent cells contribute to the tumour microenvironment (TME), specifically, the emerging evidence that such senescent cells can be detected within the stroma. Then, we describe the evidence for Sen-Mark+ cancer cells, with a particular focus on the key senescence effectors p16 and p21. We describe the current understanding of the different prognostic implications for Sen-Mark+ cancers and consider the challenges when attempting to classifying Sen-Mark+ cancer cells. Finally, we set out therapeutic opportunities to target Sen-Mark+ cancers and discuss the important considerations of such treatment regimes. The definitions of key senescence-related processes are outlined in Fig. 1.

## p16 and p21 as key senescence effectors

**Fig. 2:**
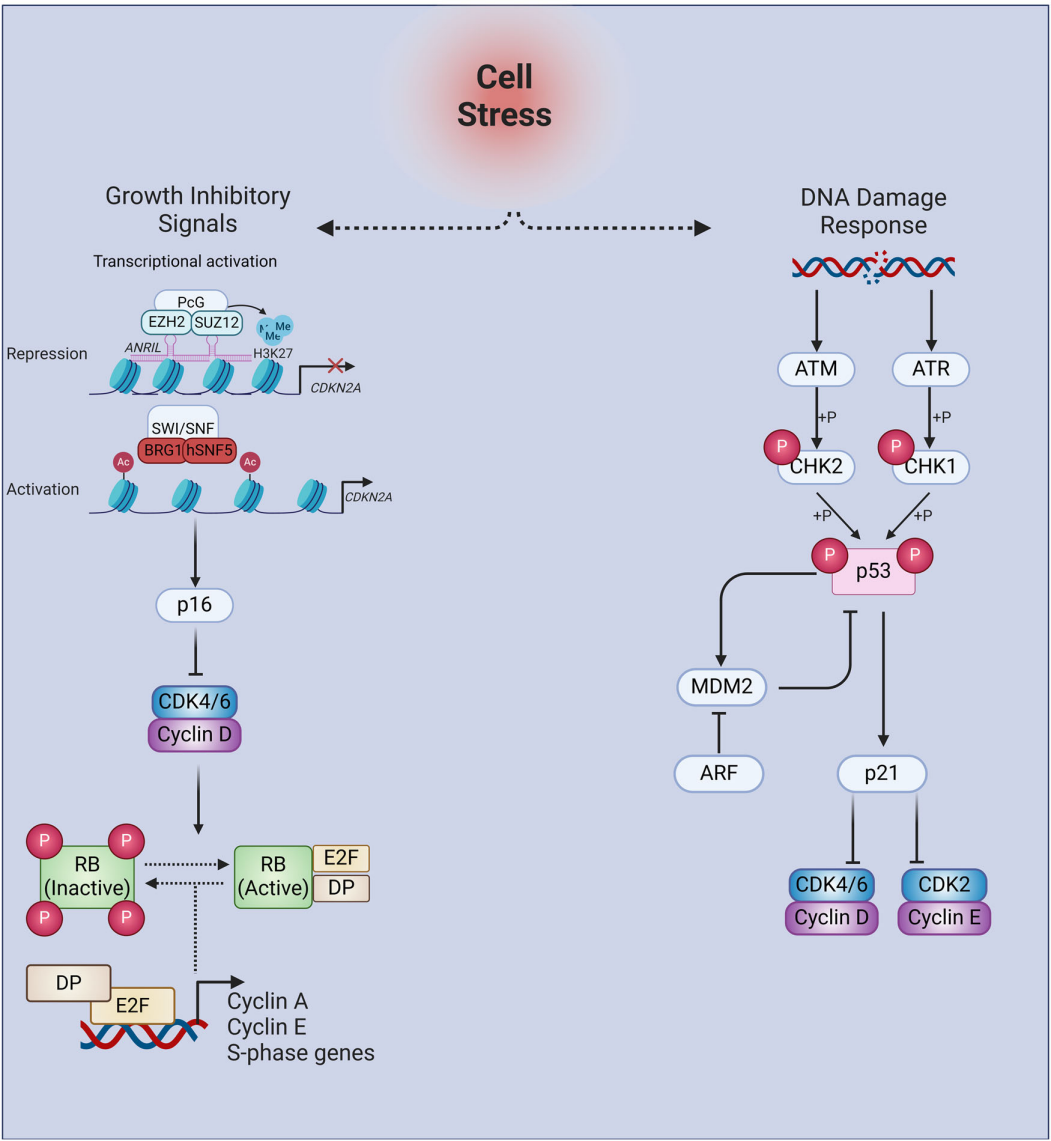
The p16/RB and p53/p21 signalling pathways in response to cell stress. Senescence inducing stimuli can result in the depression of the CDKN2A locus, allowing for the expression of CDKI p16. ANRIL, antisense lncRNA in INK4 locus, recruits PcG proteins to the CDKN2a locus. Here, EZH2, the catalytic subunit of PRC2, methylates H3K27, recruiting PRC1 to the locus and represses gene expression. The SWI/SNF chromatin remodelling complex, replaces the Bmi1 component of PRC1, exchanging histone methylation with acetylation. Catalytic subunits BRG1 and hSnf5 are essential for relaxing the histone conformation, allowing for transcription. Additionally, a range of senescence-inducing stimuli are capable of activating the DNA damage response (DDR), resulting in the stabilisation of p53 and subsequent transcriptional activation of the cyclin-dependent kinase inhibitor (CDKi) p21. Both p21 and p16 are capable of inhibiting cell cycle progression by targeting the cyclin dependent kinase/ cyclin complexes active in G1. ATM ataxia-telangiectasia mutated, ATR ataxia-telangiectasia and Rad3 related, CDK cyclin-dependent kinase, CHK1 checkpoint kinase 1, CHK2 checkpoint kinase 2, MDM2 mouse double minute 2 homolog, RB retinoblastoma protein.

The CDKi p16 and p21 play important roles as key senescence effectors. The canonical function of the tumour suppressor p16 occurs in the nucleus, during the transition of G1 to S phase of the cell cycle. Here, binding of p16 to CDK4/6 prevents the hyperphosphorylation of retinoblastoma protein (RB, pRb), maintaining the sequestration of E2F family of transcription factors, preventing S phase entry and cell cycle progression (see Fig. 2). Whilst p16 functions exclusively in the G1 to S transition, p21 can inhibit CDK4/6-cyclin D, CDK1-cyclin B1 and CDK1/2-cyclin A complexes, inhibiting the cell cycle at G1 and G2, respectively.

## Oncogene-induced senescence (OIS) as a tumour suppressor mechanism

The first evidence for OIS was published in 1997 in IMR90 fibroblasts, where the expression of an oncogenic *Ras* allele resulted in permanent G1 arrest with features of senescence^4^. Subsequently, a series of landmark studies demonstrated that OIS occurs in vivo, including in the hematopoietic compartment (T-cell)^5^, naevi^6–8^, pituitary gland^9^, lung tissue^10,11^, prostate epithelium^12–14^ and kidneys^15^.

Further evidence for the tumour suppressive role of senescence comes from the observation that senescent cancer cells are mainly detected in pre-malignant stages of cancer development (reviewed in ref. ^16^). Additionally, mutations within senescence pathways are frequently observed in cancer, enabling neoplastic cells to proliferate, and perpetuate genetic instability towards malignant transformation. For example, early studies in prostate cancer driven by *Pten* loss in mice demonstrated an upregulation of p19^ARF^ followed by p53 and p21, resulting in senescence induction^12^. Subsequent loss of *Trp53* allowed for the resumption of proliferative capacity and complete transformation of the pre-malignant cells. Similarly, high expression of oncogenic *Hras*^*G12V*^ in a mouse model of mammary tumorigenesis resulted in senescence induction in a p16-dependent manner^17^. In humans, melanocytic naevi^6^, and adenomas of both the lung^10^ and colon^18^, demonstrate high expression of the senescence marker p16 in the pre-malignant but not the malignant counterpart, reinforcing the early protective role of senescence in cancer.

## Senescence escape

Senescence escape is defined as the re-acquisition of proliferative potential following senescence induction (reviewed in ref. ^2^). This process is well documented in normal epithelial cells, occurring via a two-step process (reviewed in ref. ^19^). A first in vitro arrest, often referred to as “stasis” or “M0”, occurs via elevated p16 signalling. However, through hypermethylation of the p16 promoter, ~1 in 10^4^ cells can escape this process and re-enter the cell cycle, before entering a stable p21-dependent senescence programme following replicative exhaustion^20,21^. Of direct relevance, carcinomas are cancers which are epithelial in origin and account for up to 90% of all human cancers. Thus, senescence escape has been proposed as one potential route to carcinoma development.

In support of this model, several reports have demonstrated that senescence escape can occur through the genetic mutation of key senescence genes, for example via the loss of p16-pRb^22^ or p53-p21 signalling^12,23^, or following the reactivation of telomerase activity^24^. Furthermore, the most frequently mutated tumour suppressors in human cancers are the *INK4/ARF* locus or *p53* (reviewed in refs. ^25,26^). The *INK4/ARF* locus comprises p15^INK4b^, p16 and p14^ARF^ (Alternative Reading Frame, ARF; p14^ARF^ in humans, p19^ARF^ in mice), transcribed in alternate reading frames of the CDKN2A gene, and is subject to extensive transcriptional regulation (reviewed in ref. ^27^). Germline mutations in *p16* are seen in familial melanoma, preventing the binding of p16 to CDK4 and allowing for uncontrolled proliferation (reviewed in ref. ^28^). Although the *INK/ARF* locus encodes two tumour suppressors, alterations to p16 expression alone are far more prevalent, with impairment of ARF activity only occurring in conjunction with loss of p16 function^26^. Generally, p16 expression is lost in human cancers either through homozygous deletion or promoter hypermethylation (reviewed in ref. ^29^). The loss of p53 function confers a substantial survival advantage to pre-cancerous cells, in part due to the consequential lack of p21 expression, as p53 directly activates p21 transcription^25^ (see Fig. 2). For example, Li-Fraumeni syndrome patients are predisposed to early onset, widespread tumour development as a result of germline *p53* mutation, emphasising the protective role of p53^30^. More commonly, p53 is inactivated directly through mutations in the *p53* gene in over half of human cancers.

In summary, these observations suggest that perturbation of senescence programmes via senescence escape represents a route to cancer development. It is important to note that other possible routes exist. For example, tumorigenesis could potentially occur without a cell formally entering a stable cell cycle arrest, a process termed senescence bypass. Differentiating between senescence escape and senescence bypass is fundamentally challenging when considering cancer evolution in vivo.

## Senescence and cancer

### Non-malignant senescent cells in the TME

It is well established that cancer cells have complex, dynamic, and bidirectional interactions with neighbouring stromal cells. There is now growing evidence that non-malignant senescent cells, including fibroblasts, endothelial cells and immune cells, present within the TME can contribute to this crosstalk. The question as to how and when these cells enter senescence is one of great interest, with emerging evidence suggesting that senescent stromal cells can be detected prior to tumour development. The role of senescent stromal cells is likely underpinned by their precise secretory profile, in a TME context-dependent manner. For example, Ruhland and colleagues established an inducible p27^Kip1^ model of in vivo senescence specifically in cells of mesenchymal origin. In this model, senescent mouse fibroblasts displayed elevated secretion of the canonical SASP factor, interleukin-6 (IL-6), and co-injection of senescent fibroblasts led to an increase in *Ras*-induced tumour growth, suggesting that non-malignant senescent stromal cells can drive tumour development^31^. More recently, Prieto et al.^32^ and Haston et al.^33^ have used a *KRas-*driven mouse models of lung cancer to uncover a pro-tumorigenic role for senescent macrophages. These publications also find that macrophages with senescent features are detected in normal, aged mice and pre-malignant human lesions, raising the spectre that their presence may prime neighbouring epithelial cells for neoplastic transformation. Further, the demonstration that genetic or pharmacological removal of senescent macrophages decreased tumour burden and increased survival illuminates routes for future therapeutic innovation. Taken together, these findings illustrate that non-malignant stromal cells add a further dimension to tumour biology.

### Senescent-marker positive (Sen-Mark+) cancer cells

As detailed above, the loss of key senescence effectors has classically been viewed as an essential step in tumour development. However, of direct relevance to this review, a subset of treatment naïve human tumours express canonical senescent effector proteins whilst simultaneously, but paradoxically, maintaining a high proliferative index. Here, we focus on p16+ and p21+ cancer cells. These underexplored ‘senescent-marker positive’ (Sen-Mark+) cancer cells create challenges, including accurate classification of senescence within the tumour, and opportunities, such as the potential for diagnostic and prognostic biomarkers. They also raise the question as to whether Sen-Mark+ cancer cells could drive therapy resistance, and if their unique expression profiles can be exploited for the treatment of these subtypes of cancer.

### p16 overexpression in cancer

Although p16 function is lost in over half of all human cancers^34^, an interesting paradigm is emerging where p16 is detected and even overexpressed in malignancy. For example, in HPV-related cancers, p16 is often overexpressed as a result of the viral E7 oncoprotein preventing SUZ12 binding, thereby inactivating RB and releasing p16 from a negative feedback loop^35^. In this context, p16 expression is associated with better overall survival^36^. Although the viral E7 protein inactivates RB, mutations in *Rb1* alone are only commonly seen as a driver in retinoblastoma and small cell lung cancer, and occur as a late event in other malignancies (reviewed in ref. ^37^). This raises an interesting conundrum. If p16 function was exclusive to the RB pathway, it would be logical that inactivation of this pathway would occur through the targeting of p16 or RB. Furthermore, although the RB1 pocket proteins are structurally similar to RB, mutations are not seen in p107 (RBL1) or p130 (RBL2)^37^. Therefore, cells that develop into cancer have preferentially lost p16 function and not the p16-RB pathway alone.

The selective pressure for cancer cells to lose p16 function, and not just the p16-RB pathway, highlights the potential alternative functions p16 may have, and what other mechanisms may exist to inactivate them. For example, in a lung epithelial model the expression of p16 is protective against DNA damage induced by bleomycin treatment, even when *Rb1* is deficient^38^. Therefore, it is important for future studies to consider protective functions of p16 beyond RB and potentially beyond the pocket protein family.

Surprisingly, the overexpression of the CDKi p16 has been reported to correlate with disease progression and poorer patient outcome in some cancer subtypes. Reconsideration of the original staining criteria established by Geradts et al. in 1995^39^, has led to the inclusion of cytoplasmic p16 in defining a positive signature. This has been essential to exploring alternative functions of p16 in cancer. In a study of gastrointestinal stromal tumours (GISTs), Haller et al. utilised two monoclonal anti-p16 antibodies for both Western blot and immunohistochemistry analysis, to gain confidence in the cytoplasmic localisation of p16^40^. Patients with high cytoplasmic p16 staining had shorter overall survival time, independent of anatomic tumour site. Interestingly, the cytoplasmic localisation of p16 was found only in cancerous cells, whereas nuclear positivity was detected in non-neoplastic cells. Given the canonical CDKi function of p16 occurs in the nucleus, the prevalence of cytoplasmic staining in a variety of cancers raises questions regarding alternative non-canonical roles for p16, or how cancer cells may adapt alternative methods to enable progression through the cell cycle despite retaining a p16-positive status.

The cytoplasmic localisation of p16 is not restricted to GISTs. In ovarian cancer without HPV infection, p16 overexpression within the nucleus and cytoplasm is present in invasive serous papillary carcinoma, without the loss of RB^41^. Intriguingly, Todd and colleagues demonstrated that ectopic overexpression of p16 in p16-positive ovarian cancer cell lines did not result in a cessation of the cell cycle, despite co-expressing RB, suggesting that unidentified downstream pathway defects could be present^42^. Furthermore, Zhao et al. demonstrated cytoplasmic p16 staining in adenomas and carcinomas arising from colorectal epithelial cells^43^. A study of 194 patients with colorectal adenocarcinoma also demonstrated p16 expression in 80% of tumours, with overexpression in 48%. Similarly to GISTs, cytoplasmic p16 was associated with a more aggressive disease and reduced survival time^44^. In squamous cell carcinoma, p16 expression did not affect proliferation rates, with p16-expressing keratinocytes present at the invasive front^45^. Interestingly, invasive squamous cell carcinoma predominantly exhibited only cytoplasmic p16. Similarly, oral squamous cell carcinoma demonstrated increased expression of p16 and p53 as the disease progressed from mild dysplasia to invasive carcinoma using immunohistochemistry^46^.

Milde-Langosch and colleagues explored the expression level of p16 and RB in 60 mammary carcinomas and four cell lines in comparison to normal mammary tissue. Surprisingly, both nuclear and cytoplasmic p16 was detected in most carcinomas, with a high level of expression in 9 cases^47^. High p16 expression was not associated with a change in RB expression but associated with a high proliferative index measured by Ki67 expression, more aggressive tumours, and poorer patient prognosis. The high proliferation rate of these tumours despite expressing the potent CDKi p16 is perplexing and challenges our current understanding on the roles and regulation of p16. Furthermore, this work supports previous observations that subcellular distribution of p16 could represent an underappreciated nuance to tumour classification^48^.

Basal-like breast cancer (BLBC) is a highly aggressive and invasive subtype of breast cancer, that has been reported to overexpress both nuclear and cytoplasmic p16^49,50^. As BLBC is commonly triple negative, it lacks any targeted therapies. Recently, the overexpression of both nuclear and cytoplasmic p16 has been suggested as a biomarker of poor prognosis in BLBC^50^. Therefore, a marker usually used to denote a senescent state, here denotes the polar opposite: a highly proliferative and aggressive cancer cell.

The cytoplasmic localisation of p16 could have unexplored therapeutic benefits. Shen and colleagues explored the cytoplasmic localisation of p16 in gastric and colonic adenocarcinomas, demonstrating a cytoplasmic interaction with anion exchanger 1 (AE1)^51^. Subsequent knockdown of AE1 using siRNA, resulted in both the translocation of p16 into the nucleus and a cessation of proliferation. This work highlights that non-canonical mechanisms of p16 regulation exist that may be exploited by cancer cells to prevent the induction of senescence.

Taken together, these examples illuminate the underexplored relationship between p16 overexpression and cancer. By understanding how these cancer subtypes have managed to escape, or bypass, senescence induction without the loss of p16, not only would the field gain valuable insight into unknown regulation mechanisms and functions of p16, but also there is the opportunity to exploit this overexpression as a new therapeutic strategy.

### p21 overexpression in cancer

Although p53 is frequently mutated in cancer, p21 mutations are rare. Outside of the nucleus, alternative roles of p21 in the cytoplasm have also been reported, such as inhibiting apoptosis by forming a complex with the apoptosis signal-regulating kinase 1 (ASK1) (reviewed in ref. ^52^). Furthermore, p21 can be activated in p53-independent methods, raising interesting questions regarding non-canonical functions within a p53-mutated cell.

Similarly to p16, both loss and overexpression of p21 have been reported with conflicting effects depending on the cancer type. In gastric cancers, p21 is detected in both the nucleus and cytoplasm, with cytoplasmic p21 being associated with metastasis and reduced overall survival^53^. In HER2-positive breast cancer, a phosphorylated form of p21 is overexpressed, with high cytoplasmic localisation being predictive of poor prognosis^54^. It was therefore suggested that phospho-p21 could be used as a predictor for patient survival in HER2-positive breast cancer.

Using an array of human tumours, Galanos et al. demonstrated an association of the CDKi p21 with the proliferative marker Ki67^55^. Furthermore, Galanos explored ability of osteosarcoma cells to escape senescence, showing that p21-expressing, p53-deficient cells regained proliferative capacity after 20 days. Cells that escaped had greater genetic instability and a more invasive phenotype, despite expressing p21. This was later attributed to a switch from RAD-51 DNA repair to the more error-prone RAD52-dependent break-induced replication^56^. By understanding how these cells have escaped senescence induction, a dependency on RAD52 has been exposed which could be exploited as a synthetic lethality therapeutic.

Interestingly, p21 overexpression in cancer cells can also lead to chemotherapeutic resistance. Alpelisib (BYL719) is a PI3Kα inhibitor, therapeutically used for *PIK3CA*-mutant, ER+ metastatic breast cancer^57^. Using integrative modelling of the T47D ER+ breast cancer cell line, Yip and colleagues explored the emergence of Alpelisib-resistant pools^58^. Here, resistant cells demonstrated nuclear p21 expression but low expression of the senescent marker, senescence-associated β-galactosidase (SA-β-Gal). In this context, expression of p21 in resistant pools allowed for the repair of DNA damage and subsequent bypass of therapy-induced senescence (TIS).

Taken together, the counterintuitive finding that p16 or p21 positive cancers occur and that this signature can be predictive of poor prognosis raises the challenging question as to how these cancers arose, and if this involved escape or bypass from an original senescence programme. These observations also suggest that alternative mechanisms to inactivate senescence may exist beyond genetic mutations, deletions, or promoter methylation of key effectors of this programme^59^. However, to explore the role of senescent-positive cancer cells in tumorigenesis and the potential to therapeutically target these cells, there are several challenges that need to be addressed. An important step will be the establishment of a reliable method to accurately define senescence-positive cancer cells. The obstacles in developing such methods and the therapeutic avenues for the restoration of tumour suppressor function through pro-senescence therapy are explored below.

## Exploiting senescence as a therapy in cancer

As detailed above, senescence is a *bone fide* tumour suppressor mechanism. Thus, engaging this cellular programme in malignant cells is a targeted therapeutic concept which is gathering momentum. This is in part because of a growing number of strategies to induce senescence in cancer cells in vivo combined with the potential that senescent cancer cells could be removed either pharmacologically and/or via immunotherapy-based approaches.

### Therapy-induced senescence

Conventional first-line cancer treatments, such as chemotherapy and radiotherapy, elicit persistent DNA damage with the aim of cancer cell eradication. However, it is now appreciated that these treatments can induce “off-target” senescence at sub-lethal doses, a phenomenon named TIS. Whilst TIS could, in theory, represent an alternative beneficial outcome for anticancer therapies, lingering senescent cancer cells are associated with immune evasion, polyploidy, senescence escape and tumour relapse^60^. Indeed, persisting chemotherapy-induced senescent tumour cells have been shown to evade immunosurveillance though the upregulation of immunosuppressive checkpoint proteins including PD-L1, CD80 and PD-L2^61,62^. In this context, the combination of chemotherapy with checkpoint inhibitors could be explored to circumvent the vulnerabilities arising from TIS. Moreover, TIS results in the increased induction of polyploidy due to DNA replication without cell division in several types of cancer^63–66^. For example, Puig and colleagues showed that following treatment with cisplatin, PROb colon cancer cells initially stopped mitotic activity and expressed markers of senescence (including SA-β-Gal and increased expression of p16 and p21), but maintained DNA synthesis. These mono- or multi-nucleated cells eventually regained proliferative capacity and resisted further chemotherapeutic treatment, accounting for tumour relapse after initial efficient chemotherapy^63^. Given these and other findings, there is a growing focus on understanding the role of senescence escape in relation to TIS, to provide better treatment options and avoid relapse.

### Pro-senescence therapies

Traditional chemotherapeutics aim to target cancerous cells through the induction of DNA damage, therefore targeting cells that are rapidly dividing. Although this can be effective, the non-specific nature of chemotherapies means the side effects are severe, applying additional strain to the patients and their quality of life^67^. More recently, research has focused on developing targeted therapies which often exploit dependencies or characteristics that are specific to cancerous cells, thus not damaging normal cells.

Given the role of senescence as a barrier to cancer development, the re-establishment of a senescence programme in cancer cells, often called pro-senescence therapy, has emerged as an attractive therapeutic strategy (reviewed in ref. ^68^). Pro-senescence therapies aim to reactivate or mimic the effectors of senescence, such as p16 or p53, allowing for the intrinsic tumour mechanism to be engaged. Conceptually, this differs from TIS, where senescence induction is an “off-target effect” of a cytotoxic chemotherapeutic agent (reviewed in ref. ^69^).

To date, several groups have attempted to identify compounds with the capacity to directly induce senescence in cancer cells. By screening a synthetic compound library, a group of compounds called Nutlins were identified to prevent the inhibitory binding of MDM2 to wild type p53, leading to elevated levels of p53 and p21 and subsequent cell cycle arrest^70^. Although effective, Nutlins depend on the expression of wild type p53, which we know to be heavily mutated in human malignancies. Therefore, although these compounds are able to function in a pro-senescence capacity, this is limited to cancer subtypes with wild type p53 expression. An alternate drug screen conducted in an osteosarcoma cell line with a mutant p53 protein, identified a compound named PRIMA-1 that was able to restore DNA binding capability to mutant p53, resulting in apoptosis and tumour suppression when tested in vivo^71^. PRIMA-1 therefore has an advantage over Nutlins and could be used to induce senescence in cancers where p53 is mutated.

Impairment of the G1/S restriction point through the loss of p16 function imparts a profound proliferative advantage to cancer cells. To circumvent this, pyridopyrimidines that had previously been shown to inhibit CDKs, were screened to find a compound capable of inducing a G1 arrest through the inhibition of CDK4-cyclin D activity^72^. The discovered cytostatic compound, PD-0332991 or Palbociclib, demonstrated antitumour efficacy when tested against tumour xenograft models. In 2015, Palbociclib was licenced for treatment of oestrogen positive (ER+), HER2-negative breast cancer in combination with the aromatase inhibitor letrozole, to lower oestrogen levels^73^. Two further CDK4/6 inhibitors have subsequently been discovered, Abemaciclib and Ribociclib^74^. Although CDK4/6 inhibitors show benefit in a subset of cancers, resistance to therapy can arise through spontaneous mutations in RB1^75^ or the overexpression of CDK6^76^. Interestingly, high p16 expression in ER+ breast cancer patient-derived xenografts resulted in resistance to Ribociclib^77^. Of these resistant tumours, 17% had normal levels of RB suggesting that high levels of p16 alone are predictive of resistance. It has also been demonstrated that prolonged treatment with CDK4/6 inhibitors can result in senescence in stromal fibroblasts, resulting in pro-tumorigenic SASP secretion^78^.

### Senolytics

Senolytics are a class of molecules that selectively eliminate senescent cells (reviewed in refs. ^79,80^). These were developed following seminal work demonstrating that accumulation of senescent cells contributes to a range of age-related pathologies and that selective senescent cell clearance alleviates these effects^81^. Early senolytics included combination treatment with Dasatanib and Quercertin, which have been successfully used to limit age-related functional decline in mice^82,83^. However, senolytic activity has proven to vary widely in efficacy between different senescent contexts and the list of senolytics continues to grow, including cardiac glycosides, and perhaps the most widely used BCL-2 inhibitors such as Navitoclax^84–86^.

The relevance to cancer comes from an innovative approach from Wang et al., who propose a “one-two punch” method to cancer cell clearance^87^. The first punch involves the pharmacological activation of senescence using pro-senescence approaches. This is then followed by a second senolytic punch, facilitating the selective clearance of the now senescent cancer cell. This approach generates the benefit of senescence induction in the context of cancer, whilst avoiding the potential deleterious effects of senescent cell accumulation^67,88^. However, it relies on the availability of both a reliable means of senescence induction in the required cancer model, as well as a paired effective senolytic. As a therapeutic strategy, it may be preferable to find a senescent cancer cell selective senolytic, due to the homeostatic role of senescent cells in other tissues including roles in wound healing and development^89^. The second punch could also be beneficial following routine chemotherapeutic treatment to circumvent the challenges that TIS poses both within cancer cells and the stroma.

Furthermore, concerns have been raised about tissue loss associated with senescence clearance, particularly in aged individuals where senescent cells accumulate and might contribute to the tissue structural integrity. In this context, the use of senomorphic compounds to inhibit or modulate the pro-tumorigenic SASP might be more appropriate.

### Immunotherapy based approaches

The interaction between senescent cells and the immune system is complex, primarily due to the varied SASP profile that emerges between different senescence triggers and cell types. The SASP has been implicated a means of senescent cell clearance, with factors such as IL-8 facilitating the recruitment of innate immune cells including NK cells, macrophages, and neutrophils^90,91^. In the context of cancer, in a KRAS-mutant lung cancer patient-derived xenograft model, coupling of a mitogen-activated protein kinase inhibitor and a pro-senescence CDK4/6 inhibitor, impaired tumour growth to a greater extent than either drug alone^92^. A mouse model of lung cancer (*Kras*^*G12D/+*^*;Trp53*^−/−^) was used to further demonstrate that combination therapy resulted in enhanced immune clearance of the tumour by NK cells. This immune mediated clearance was reduced when the SASP was diminished through NF-kB knockdown. Therefore, the induction of senescence in a cancerous cell has the potential to alter its secretome, resulting in pro-inflammatory factors being secreted and provides an opportunity for immune-mediated clearance. However, the SASP has also been demonstrated to have an immunosuppressive effect^31^. Understanding the balance between positive and detrimental roles of the SASP will be essential to the design of senescence based therapeutic intervention.

### Adaptive immunity

More recently, characterisation of the senescence “surface-ome” has provided insight into the engagement of an adaptive immune response by senescent cancer cells, through identification of an upregulation of the major histocompatibility complex I (MHC-I). This, in combination with the development of a pro-immunogenic immunopeptidome, makes senescent cancer cells sensitive to cytotoxic clearance via CD8 + T cells. Excitingly, this senescence-driven adaptive immunity also facilitated the clearance of non-induced cancer cells in vivo, when engaged through either a prophylactic or treatment based “senescent cell vaccination”^93,94^. This opens new avenues for therapeutic development, with implications in both cancer immunotherapy and the instigation of a “senolytic” adaptive immune response.

## Challenges for senescence classification in cancer

**Fig. 3:**
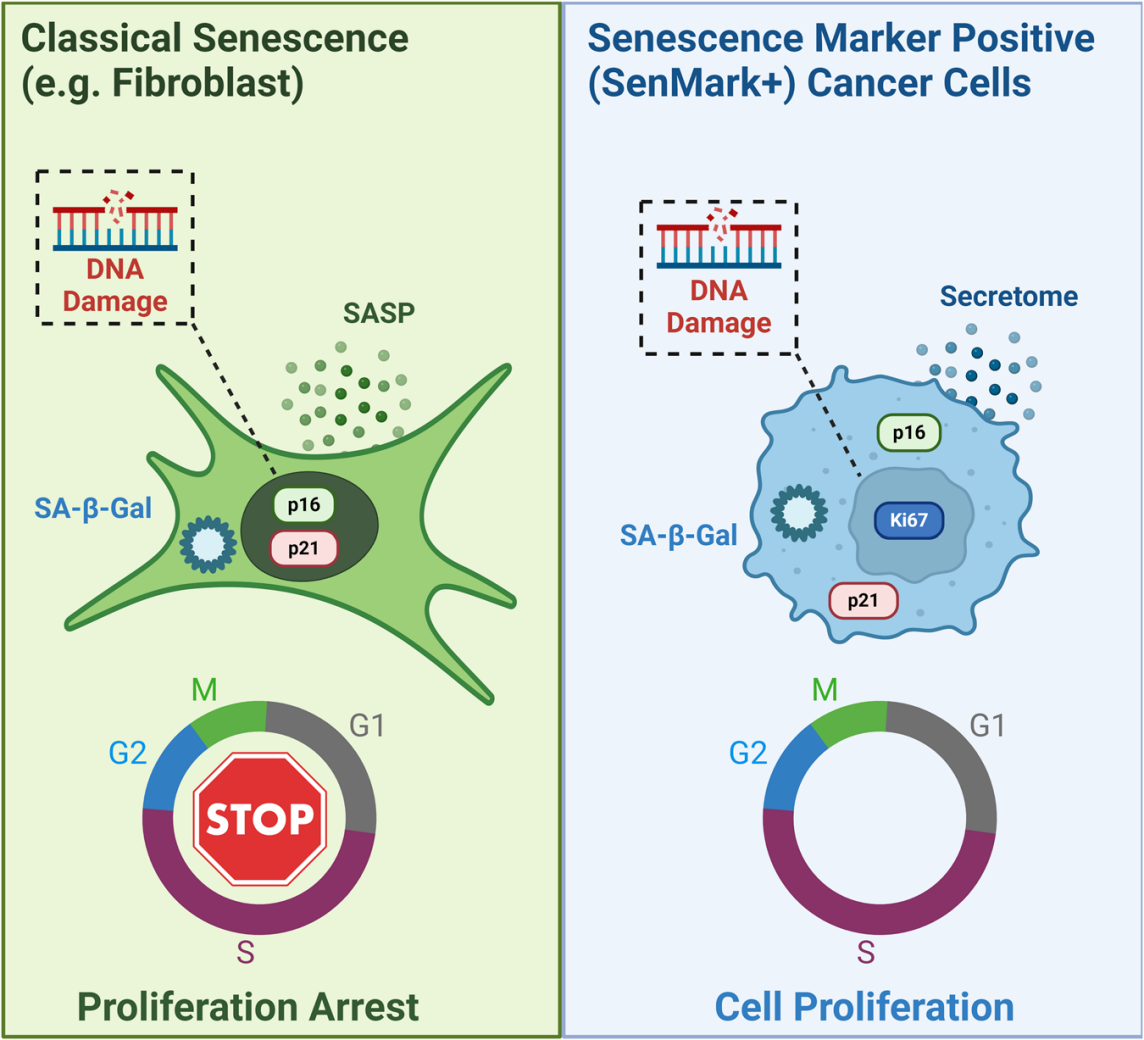
Challenges for senescence classification in cancer. Hallmarks of a classical senescence response typically involve cessation of the cell cycle, increased expression of cyclin-dependent kinase inhibitors (e.g. p16, p21), increased markers of DNA damage, increased lysosomal activity (e.g. SA-β-Gal) and secretion of a senescence-associated secretory phenotype (SASP). However, these markers are frequently present in SenMark+ cancer cells, limiting their utility as identifiers of senescence, which thus represents a significant technical challenge.

*Prima facie* senescence and cancer represent diametrically opposed cell fates that might intuitively be anticipated to be distinguishable with relative ease. However, as detailed above, non-malignant senescent cells can be detected within the TME, Sen-Mark+ cancer cells define a subset of treatment naïve tumours, and emerging therapeutic strategies aim to establish senescence in cancer cells. Taken together, classification of these three categories of cell types can be technically challenging, due to the context dependent presence or absence of canonical senescence hallmarks (Fig. 3). This is exacerbated by broader issues facing the senescence field; that there remains no universal marker of senescence and that variability exists between contexts of different cell types, inducers and tissues (reviewed in ref. ^95^). Consequently, both individually and in combination, senescence markers that are considered canonical may be misleading or lack utility when used as a means of senescence identification in cancer.

### Tumour suppressors

The critical role of tumour suppressors (p53-p21 and p16) in restraining the development of malignancy means that the failure of these pathways is generally a prerequisite of cancer development^96^. This can occur through mutation of the tumour suppressors themselves, either leading to total knockout or heterozygous mutations that lead to dominant negative functional repression (e.g. p53)^25^. Alternatively, the pathway may be disrupted downstream of the tumour suppressor, as has frequently been observed in the reciprocal RB-null state of many p16 positive cancers (reviewed in ref. ^97^). Consequently, these cancer cells are positive for these widely relied upon senescence markers but avoid their tumour suppressive role^98^. This makes identifying senescence in Sen-Mark+ cancers particularly challenging, an issue with which the field is still grappling.

### Proliferation

The loss of cellular proliferation is an intrinsic feature of senescence that provides an obvious distinction from the uncontrolled cell division associated with cancer^99^. By definition, if a cell is proliferating, it cannot be senescent. In the context of Sen-Mark+ cancer cells (e.g. p16+ or p21+ cells), these cells will be positive for both these canonical CDKi *and* proliferation. When considering pro-senescence therapies, proliferation rates are generally assessed across a population of cells within an in vitro condition, and the potential for mixed populations within cell culture conditions makes the use of proliferation problematic as a classifier of senescence alone. This is particularly the case with senescent cancer cells, as the high proliferative rate of non-senescent cancer cells can quickly overtake the population^100^. Therefore, experimental conditions must be carefully optimised to determine an appropriate endpoint where senescence can be detected^101^.

This disparity between proliferation rates is a particular issue in the context of high-throughput screening, where identifying senescence conditions within negative selection screens (e.g. via loss of proliferation) is hindered by low signal-to-noise ratios^102^. Utilisation of final cell numbers to determine proliferation loss is also limited due to similar readouts occurring through toxicity and cell death. This can be mitigated to a degree through the use of proliferation markers, such as EdU incorporation or Ki67 positivity, to demonstrate the final population contains arrested cells, as opposed to a recovering population that has avoided toxicity^103^. Therefore, whilst it is crucial to observe a loss of proliferation when attempting to classify senescent cancer cells, this is insufficient if in isolation^101^. Generally, it is accepted best practice to couple loss of proliferation with additional senescence hallmarks to construct a panel of established markers^95^. However, as detailed below, conforming to this blueprint is challenging in many cancer contexts.

### Other markers

A wide range of senescence markers have been proposed, which usually require tailoring to the specific context of the model system under investigation. However, as in all fields, some have proven more popular than others, and are seen as the “go to” for senescence classification^95^. Increased SA-β-Gal activity is one of the longest-standing markers of senescence and represents a useful assay which exploits the enhanced lysosomal content generally associated with senescent cells^104^. However, the SA-β-gal marker has proved to be limited, with comparable activity observed in quiescent cells^105^. Furthermore, cancer cells often also have increased lysosomal levels and frequently appear “positive” for SA-β-gal^106^. This can be the case whether the cancer cell is Sen-Mark+ or not, and as an assay has the potential to generate false positives. The assay itself is also notorious for requiring careful optimisation and is sensitive to considerations of cell confluency^105,107^. Therefore, as a means of distinguishing between proliferating and senescent cancer cells, it must be employed with caution.

Another widely utilised marker is the assessment of features of DNA damage^108^. This is most relevant in contexts where senescence induction occurs through relevant stimuli, such as exposure to ionising radiation or cytotoxic compounds. However, many cancers are also associated with the DNA damage response pathway, so standard markers, such as γH2AX staining, in isolation are insufficient to classify a cancer cell as senescent^109^.

The acquisition of the SASP is an extremely well-established hallmark of senescence^110^. However, the SASP has been shown to be highly context-dependent, varying across triggers and cell types, as well as dynamically developing across the course of senescence induction^111^. In this, it reflects the challenges of senescence hallmarks in general. Recently, it was demonstrated that the principal determinant of SASP factors associated with senescent cancer cells is the parental cell itself^112^. Therefore, it would be a challenge to rely upon any individual or combination of secreted factors to confidently classify cancer cells as senescent^113^. It is important to emphasise that often senescence markers have been assessed in the context of non-malignant cells (mainly fibroblasts). Therefore, it is perhaps unsurprising that markers of senescent epithelial cancer cells themselves are less developed. However, recent efforts within the field have begun to redress that balance.

### Overcoming the challenge with machine learning

**Fig. 4:**
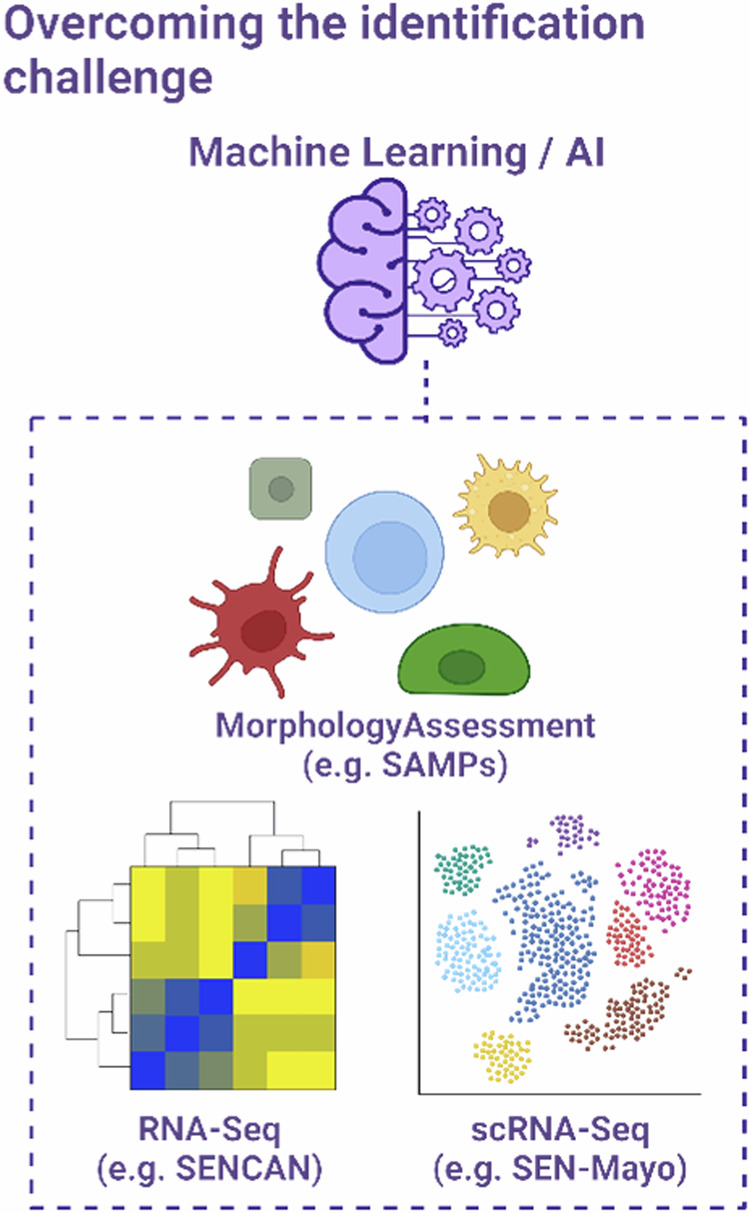
Senescence classification with machine learning. With increasingly large and complex datasets, sophisticated machine learning algorithms represent a potential route to overcoming the challenges of senescence identification. These have been applied to develop classifiers based on a diverse range of data types including multimetric morphology imaging data, RNA-sequencing or sc-RNA-sequencing.

A major new tool in the repertoire of researchers aiming to identify senescent cancer cells is the increasing accessibility of machine learning (ML) algorithms^114,115^ (Fig. 4). These have recently been employed to demonstrate that senescent cancer cells can be distinguished from their proliferating counterparts through image-based morphological analysis^115^. Senescence-associated morphological phenotypes (SAMPs) can be extracted from simple microscopy images and facilitate the exploration of both the heterogeneity between senescence contexts, and within an individual model, through the use of single target data and dimensionality reduction algorithms (e.g. tSNE/UMAP). These morphological profiles have recently been used to develop a feature-based classification tree for the identification of senescence in vitro and in vivo^107^. This work supports developments employing convolutional neural network deep learning methods, to generate computer vision-based tools of senescence classification^116,117^.

Collectively, these studies support a principle that dates to the earliest days of the senescence field, that senescent cells are morphologically fundamentally different from their proliferating counterparts^118^. Importantly, the ML methods employed in these studies were found to recapitulate in vivo, which has often not been the case for many canonical in vitro senescence hallmarks^107,115^. Application of sophisticated image analysis methodologies allows an increasingly complex assessment of morphological parameters, and the field of high throughput phenomics has potential to overhaul the requirements of senescent cell classification, particularly in contexts where the use of canonical markers is logistically restricted (e.g. screening) or biologically limited (Sen-Mark+ cancers and senescent cancer cells)^114^.

Machine learning has also been employed to provide insight to the increasingly complex datasets being generated by the senescence field. Jochems et al. developed the SENCAN classification model, which was constructed through bulk RNA sequencing data from 13 cancer cell lines induced to senescence via treatment with two small molecules^112^. SENCAN is an elastic net-based classifier, which identified 137 genes that collectively can be used to predict senescence, expanding the genes of interest beyond what might be considered “standard” senescence markers. Intriguingly, whilst SENCAN was trained with data from senescent cancer cells, it was able to correctly classify both senescent fibroblast and HUVEC cells, suggesting that a combination of ML and RNA-seq may be a route towards the so far elusive “universal” marker of senescence. However, the failure of SENCAN to classify non-malignant senescent aortic endothelial cells, emphasises that this remains a distant achievement for the field given the wide range of senescence contexts^101^.

The scale of this heterogeneity seems likely to continue expanding, especially due to the increased use of single-cell RNA sequencing, which allows insight within an individual senescence model. A notable example of this is the “SenMayo” classification tool, which (similar to SENCAN) employs the assessment of 125 genes to predict a senescence state^119^. Whilst this system was developed in the context of ageing, similar tools in the context of cancer could build on the groundwork of SENCAN to develop more robust predictors of senescence. Ultimately, as such tools require training on a wide range of data sets, endeavours such as the SenNet consortium and the construction of large atlases of senescent cell data will be invaluable to this^120^.

## Future perspective

The overexpression p16 and p21 in a subset of cancers is a perplexing paradox, where in some situations the expression of a CDKi is counterintuitively linked to poor patient outcome. Furthermore, the high expression levels are often predictive of resistance to therapy. Interestingly, it appears that the localisation of these CDKi is important, with cytoplasmic expression of either p21 or p16 being associated with reduced overall survival. The localisation of these proteins raises interesting questions regarding alternative functionality and regulation they may have given the ability of the cancer cells to escape, or bypass, senescence induction. Pro-senescence therapies are an exciting area of ongoing research, aiming to specifically induce senescence in cancer cells, to allow the intrinsic tumour suppressor mechanism to take effect. This can also be coupled with senolytics, in a one-two punch strategy^67^. An alternative approach could involve the re-sensitisation of cancer cells to their endogenous overexpression of CDKi, p16 and/or p21. To fully elucidate the therapeutic potential of re-sensitisation of senescent-positive cancer cells, more research is required to understand the potential non-canonical role of CDKi in the context of different cellular localisation. Compounds facilitating localisation of CDKi to the nucleus could provide a previously unappreciated mechanism to induce pro-senescence in a cancer-specific manner.

The observation of that cancer cells have the potential to proliferate and drive tumour development while retaining key senescent proteins, such as p16, raises intriguing questions about the mechanisms underlying cancer progression. Additionally, the varied cellular localisation of p16 in different cancer types underscores the need to explore alternate roles and regulation mechanisms of p16 that may be exploited by cancer cells to evade senescence induction. Furthermore, the intricate interplay between p16 and other tumour suppressors, including p53 and p21, adds another layer of complexity to our understanding of cancer biology. The subcellular localization of p16 and p21, for instance, has been associated with differential prognostic implications in various cancer types. However, challenges with the identification of senescent cancer cells remains an outstanding issue, particularly in the context of “senescence marker positive cancer cells”. Overcoming these challenges will be essential to developing new targeted pro-senescence-based therapies. Overall, elucidating the molecular mechanisms underlying CDKi overexpression in cancer and their interactions with tumour suppressors holds promise for the development of therapeutic strategies and prognostic markers in cancer management. Continued research in this field is essential to unravel the full spectrum of p16 and p21 functions and thus the implications for cancer biology and therapy.
